# 2D MXene: A Potential Candidate for Photovoltaic Cells? A Critical Review

**DOI:** 10.1002/advs.202104743

**Published:** 2022-02-15

**Authors:** Muhammad Ahsan Saeed, Asif Shahzad, Kashif Rasool, Fahad Mateen, Jae‐Min Oh, Jae Won Shim

**Affiliations:** ^1^ Division of Electronics and Electrical Engineering Dongguk University Seoul 04620 Republic of Korea; ^2^ Department of Energy and Materials Engineering Dongguk University Seoul 04620 Republic of Korea; ^3^ Qatar Environment and Energy Research Institute Hamad Bin Khalifa University (HBKU) Qatar Foundation 34110 Doha Qatar; ^4^ Department of Chemical and Biochemical Engineering Dongguk University Seoul 04620 Republic of Korea; ^5^ School of Electrical Engineering Korea University Seoul 02841 Republic of Korea

**Keywords:** interfacial layers, MXene, photovoltaic cells, stability, titanium carbide, transparent conductive electrodes

## Abstract

The 2D transition metal carbides/nitrides (2D MXenes) are a versatile class of 2D materials for photovoltaic (PV) systems. The numerous advantages of MXenes, including their excellent metallic conductivity, high optical transmittance, solution processability, tunable work‐function, and hydrophilicity, make them suitable for deployment in PV technology. This comprehensive review focuses on the synthesis methodologies and properties of MXenes and MXene‐based materials for PV systems. Titanium carbide MXene (Ti_3_C_2_T*_x_*), a well‐known member of the MXene family, has been studied in many PV applications. Herein, the effectiveness of Ti_3_C_2_T*_x_* as an additive in different types of PV cells, and the synergetic impact of Ti_3_C_2_T*_x_* as an interfacial material on the photovoltaic performance of PV cells, are systematically examined. Subsequently, the utilization of Ti_3_C_2_T*_x_* as a transparent conductive electrode, and its influence on the stability of the PV cells, are discussed. This review also considers problems that emerged from previous studies, and provides guidelines for the further exploration of Ti_3_C_2_T*_x_* and other members of the 2D MXene family in PV technology. This timely study is expected to provide comprehensive understanding of the current status of MXenes, and to set the direction for the future development in 2D material design and processing for PVs.

## Graphical abstract

Recent progress on MXene‐based PVs is discussed. Synthesis procedures and properties of MXene are presented. The role of MXene as an additive, interfacial layer, and as a conductive electrode in PVs is comprehensively studied. Later, the influence of MXene on the stability of PVs is described. Finally, guidelines to overcome the challenges of MXene based materials for PVs are provided.

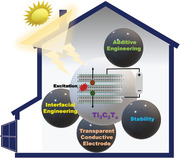

## 1 Introduction

Over the past few years, the development of crucial generators of clean energy that are economically viable has become a global challenge because of the rapid increase in the energy demand in our daily lives. Fossil fuels, which despite the rapid diminishment of oil reserves, satisfy more than 85% of that energy demand, produce environmental pollutants and greenhouse gases that contribute to global warming.^[^ ^1^, ^2^, ^3^ ^]^ Scientists are continuously searching for alternative renewable and cost‐effective energy resources that include wind, thermal, solar, and hydro energy to address the aforementioned environmental problems.^[^ ^4^, ^5^, ^6^ ^]^ Among these resources, because of the abundance, cost‐effectiveness, and environmental friendliness of sunlight, and the fact that it provides an endless supply of power, photovoltaic (PV) technology has become a major energy provider.^[^ ^7^, ^8^, ^9^, ^10^, ^11^, ^12^ ^]^ In this regard, significant attention has been devoted to the development of novel nanostructured materials to improve PV performance and reduce the cost of power generation. Emerging materials, including 2D nanomaterials, organic polymers, and oxides, have been widely used in the energy conversion processes for PV technologies.^[^ ^13^, ^14^, ^15^, ^16^, ^17^, ^18^, ^19^, ^20^ ^]^

Among these materials, Gogotsi et al. in 2011 reported 2D MXene nanosheets based on transition metal carbide and nitride materials.^[^ ^21^ ^]^ These materials have been extensively studied for numerous applications that include sensors,^[^ ^22^, ^23^, ^24^ ^]^ light‐emitting diodes,^[^ ^25^, ^26^, ^27^ ^]^ energy storage,^[^ ^28^, ^29^, ^30^, ^31^, ^32^, ^33^, ^34^ ^]^ water purification,^[^ ^35^, ^36^, ^37^, ^38^ ^]^ supercapacitors,^[^ ^39^, ^40^, ^41^, ^42^ ^]^ photocatalysis,^[^ ^43^, ^44^, ^45^, ^46^ ^]^ biomedical,^[^ ^47^, ^48^, ^49^, ^50^, ^51^, ^52^ ^]^ and electromagnetic^[^ ^52^, ^53^, ^54^, ^55^ ^]^ applications. MXenes are a unique and unusual combination of early transition metal carbide/nitrides or carbonitrides that are typically synthesized by a top–down selective etching procedure of their parent hexagonal MAX phases.^[^ ^21^ ^]^ MAX phases are layered ternary compounds with the general formula M*_n_*_+1_AX*_n_* (*n* = 1, 2, 3, or 4), where M represents the early transition metals (V, Ti, Mo, Zr, etc.) in the periodic table of elements;^[^ ^56^, ^57^, ^58^ ^]^ A represents group A elements (Al, Cd, Si, Pd, Ga, etc.); and X may be carbon, nitrogen, or a mixture of these two elements.^[^ ^59^ ^]^ Consequently, MXenes are 2D inorganic compounds that consist of carbides, nitrides, and carbonitrides,^[^ ^60^ ^]^ which are synthesized from parent M*_n_*_ + 1_AX*_n_* (MAX) precursors (e.g., Ti_3_AlC_2_).^[^ ^61^ ^]^ The MXene surface is terminated by functional groups that are typically represented by T*_x_* (e.g., Ti_3_C_2_T*_x_*), and the hydrophilic nature of MXenes is attributed to the presence of hydroxyl‐ or oxygen‐terminated surfaces.^[^ ^35^ ^]^ Owing to their unique structural, electrical, and chemical properties, they are used in several applications, which include PV technology.

Recently, MXenes have shown promise for use in PV technology owing to their unique optoelectronic properties, such as their large charge carrier mobility, excellent metallic conductivity, high optical transmittance, and tunable work function (WF).^[^ ^62^, ^63^, ^64^ ^]^ Ti_3_C_2_T*_x_* (titanium carbide) is a representative member of the versatile 2D MXene family, and has been utilized in many applications that include PV technology.^[^ ^65^ ^]^ The rich chemistry and uniform surface termination of Ti_3_C_2_T*_x_* offer vast possibilities for tuning their electrical properties. Additionally, the synthesis of MXenes enables their surfaces to be naturally functionalized, which modifies the electrostatic potential near the surface, leading to a shift in the WF of the thin films.^[^ ^66^, ^67^ ^]^ Ti_3_C_2_T*_x_* was used in MAPbI_3_‐based perovskite solar cells for the first time in 2018, and since then, it has found application in conductive electrodes,^[^ ^68^ ^]^ as an additive in the photoactive layer,^[^ ^69^, ^70^ ^]^ and in the interfacial layers of solar cells.^[^ ^71^ ^]^ The growing emergence of the incorporation of MXenes in PV cells necessitates a systematic and timely study to comprehend the fundamental physical processes involved in the improvement of the power conversion efficiency of solar cells.

From this perspective, this review summarizes recent progress in PV cells based on MXene materials. Based on previous studies, the synergistic role of MXene in PVs is explored by illustrating the design for their integration in PV technology. Subsequently, a comprehensive discussion of the effectiveness of MXene as an additive in PV cells is presented. Additionally, the performance of MXene‐PV cells is examined in‐depth in the context of interfacial layers, transparent conductive electrodes, and stability. Finally, an analysis is provided of existing issues that limit the device performance, along with a critical perspective for the development of new MXenes for photovoltaics.

## 2 Synthesis and Characteristics of MXene

### 2.1 Exfoliation of MAX Phase and the Delamination of MXene

**Figure 1:**
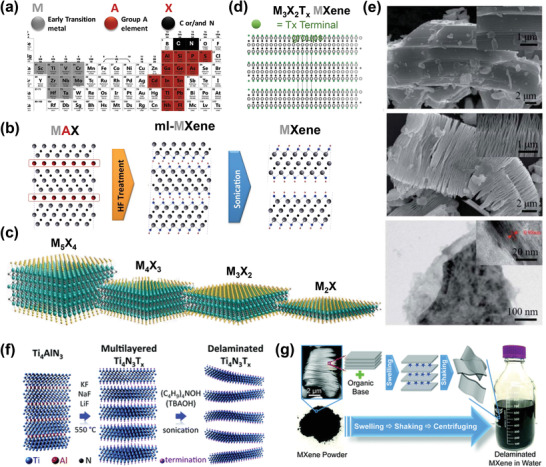
a) Periodic table of the elements showing the possible stoichiometric combination of MAX phases: an early transition metal, group A element, and carbon, nitrogen, or both, can formulate the MAX phase. b) Schematic of the etching process of the MAX phase and the formation of MXene, and c) graphical illustration of the different MXenes represented by the general formula M*_n_*_+1_X*_n_*. a,b) (Reproduced with permission.^[^ ^21^ ^]^ Copyright 2011, Wiley‐VCH. c) Reproduced with permission.^[^ ^75^ ^]^ Copyright 2021, Wiley‐VCH. d) Schematic of M_3_X_2_T*_x_* MXene, for example, Ti_3_C_2_T*_x_*, where T*_x_* represents surface terminal groups, such as O, OH, F, and Cl. e) SEM images of Ti_3_AlC_2_ MAX phase, ml‐Ti_3_C_2_T*_x_*, and TEM image of ml‐Ti_3_C_2_T*_x_*. Reproduced with permission.^[^ ^91^ ^]^ Copyright 2019, Royal Society of Chemistry. f) Ti_4_AlN_3_ exfoliation into ml‐Ti_4_N_3_T*_x_* MXene by molten salt treatment, and then exfoliation into dl‐Ti_4_N_3_Tx MXene. Tetrabutylammonium hydroxide was used as an intercalating agent to widen the interlayer distance of layers, which weakens the interaction between the layers, and then delaminated by sonication. Reproduced with permission.^[^ ^77^ ^]^ Copyright 2016, Royal Society of Chemistry. g) Organic compound intercalation widens the interlayer spacing and causes swelling of the structure, which can be further delaminated by shaking, and collected by centrifugation. Reproduced with permission.^[^ ^88^ ^]^ Copyright 2015, Royal Society of Chemistry.

MAX phases are a family of layered ternary transition metal carbides, and/or nitrides. MAX phases are unique and unusual combinations of metals and ceramics with a highly anisotropic hexagonal crystal structure.^[^ ^72^ ^]^ The general chemical formula used to symbolize MAX phases is M*_n_*_+1_AX*_n_*. **Figure** **1**a shows that in the periodic table of elements, early transition metal atoms (M) and carbon/nitrogen (X) form octahedral edge‐sharing blocks (MX) interlaying with A‐element layers, and could form various stacking arrangements known as subgroups in MAX phases, which are represented as M_2_AX, M_3_AX_2_, M_4_AX_3_, and M_5_AX_4_.^[^ ^73^ ^]^ After years of research, in the mid‐90s, Barsoum and El‐Raghy synthesized for the first time a titanium silicon carbide phase (Ti_3_SiC_2_) by a simple heat sintering process, and named it the 312 MAX phase.^[^ ^74^ ^]^ More than 70 combinations of MAX phases are possible, with Ti_3_AlC_2_ being one of the most promising and studied MAX phases. MXene can be produced by eliminating the A atom from MAX phases; the resulting material is generally represented as M*_n_*_+1_X*_n_* T*_x_* (Figure 1b), where M is an early transition metal atom, X can be carbon and/or nitrogen, *n* can be 1, 2, 3, or 4, and T*_x_* is the surface terminal groups that appear after the removal of A atoms. Thus, after removal of the A layer from the MAX phases, MXenes are generally categorized as M_2_X_1_, M_3_X_2_, M_4_X_3_, and M_5_X_4_ phases (Figure 1c).

Because of the strong bonding between M and A atoms, the direct mechanical exfoliation of MAX phases into MX layers is not possible.^[^ ^75^ ^]^ However, the metallic M–A bonds in the M*_n_*_ + 1_AX*_n_* phase are chemically more active than ionic and/or covalent M–X bonds; Figure 1b shows that the selective etching of the A layer by chemical or electrochemical methods is therefore quite possible.^[^ ^73^ ^]^ In this regard, various etching procedures, including fluorine‐containing acids, such as HF or HCl+LiF,^[^ ^21^, ^76^ ^]^ molten salt,^[^ ^77^ ^]^ alkaline hydrothermal treatment,^[^ ^78^, ^79^ ^]^ electrochemical etching using HCl or NH_4_Cl/TMAOH,^[^ ^80^, ^81^ ^]^ and etching in Lewis acids, such as ZnCl_2_, can be employed.^[^ ^82^ ^]^ Wet chemical treatment using HF or HCl+LiF is the most popular and efficient etching procedure to improve the exfoliation of MAX phases. In wet etching, the HF reaction with the MAX phase, for example, the Ti_3_AlC_2_ MAX phase, is a thermodynamically favorable reaction, and HF can dissolve the Al layer, forming AlF_3,_ and produce Ti_3_C_2_ (Figure 1d). Furthermore, the etching process proceeds via a series of reactions, resulting in 2D Ti_3_C_2_T*_x_* multilayer nanosheets. Based on the etching conditions, various moieties, including Ti_3_C_2_(OH)_2_ and Ti_3_C_2_F_2_, were formed after the HF etching process, because of the formation of surface terminal groups (e.g., —F, —O, and —OH) (Figure 1c).^[^ ^83^ ^]^ In scanning electron microscopy (SEM) observations, multilayer nanosheets exhibited an accordion‐like structure, an indication of the successful etching of Al layers from the Ti_3_AlC_2_ MAX phase, which was further confirmed by energy dispersive spectroscopy (SEM‐EDS) (Figure 1e).^[^ ^84^ ^]^ However, because of their minimal etchability and selectivity, poor and unstable 2D structure formation, low‐quality flakes of 2D MXene, and requirement for high temperature in the etching process, exfoliation methods other than wet etching are not considered beneficial. For example, in the case of a molten fluoride salt treatment, a temperature of 550 °C is required to exfoliate Ti_4_AlN_3_ to produce Ti_4_N_3_ MXene (Figure 1f).^[^ ^82^, ^84^ ^]^

MXene produced by HF treatment exhibits accordion‐like multilayer MXenes structures, which can be further delaminated by a mechanical process;^[^ ^85^, ^86^ ^]^ however, intercalation of a compound can catalyze the delamination process to separate the nanosheets. A suitable solvent, for example, large organic compounds, including dimethyl sulfoxide, n‐butylamine, tetrabutylammonium hydroxide, urea, or hydrazine, can be used (Figure 1g).^[^ ^75^, ^86^, ^87^, ^88^ ^]^ The liquid delamination of MXenes in organic solvents enables the large‐scale production of MXenes, which can be used for numerous applications that include catalysis, energy storage devices, reinforcements in polymers, and photovoltaic cells.^[^ ^88^, ^89^ ^]^

### 2.2 Structural, Chemical, and Electronic Properties of MXenes

MXenes possess fascinating structural, electronic, and chemical properties. Their excellent properties, such as high electrical conductivity, mobility, surface functional groups, and tunable chemistry, have drawn significant attention in PV cell applications.^[^ ^69^ ^]^ In MXene structures, modeling of MXenes is a key tool for understanding the structural changes they undergo under altering physical conditions.^[^ ^92^ ^]^ Studies predicted that the surface terminal groups may be located above the hollow sites between the three nearby C atoms. Nonetheless, later studies indicated that the location and orientation of these functional groups are more complex than expected.^[^ ^92^, ^93^, ^94^ ^]^ MXene species and their constituent materials are responsible for their exact configuration. MXenes are usually modeled with uniform terminating species, which are not representative. HF‐based exfoliation of MXene from the MAX phase results in OH, O, and F surface‐terminated species in the real state; and upon rinsing and/or storing of MXene in water, OH replaces F, which is why OH‐ and O‐terminated groups are considered the most stable terminal groups. Xie et al. also found that during metal adsorption and/or the high‐temperature treatment of MXene, OH groups were converted into O terminal atoms.^[^ ^95^ ^]^

Chemically, MXene flakes are not stable in an oxygenated environment, or when exposed to light, which may accelerate the oxidation of the colloidal solution, and result in the formation of metal oxide nanocrystals present on the sheet edges. For example, in Ti_3_C_2_T*_x_* MXene, the formation of anatase TiO_2_ nanocrystals might occur, owing to oxidation in pressurized water, CO_2_, or air.^[^ ^96^ ^]^ However, degassed water or dry air could be suitable options for the storage of single MXene flakes for a longer period.^[^ ^73^ ^]^ Different anatases can be produced in different oxidation regimes.^[^ ^97^ ^]^ The MXene oxidation process was studied by Ghassemi et al. using in situ TEM analysis.^[^ ^98^ ^]^ In air, O_2_ reacts with Ti_3_C_2_T*_x_* to form TiO_2_, which can be the anatase or rutile phase. The distribution of newborn TiO_2_ nanocrystals on the surface of 2D Ti_3_C_2_T layers was found with a distinctive hybrid structure of TiO_2_—C. Additionally, with heat treatment, anatase TiO_2_ forms later on at the high‐temperature anatase phase, and can transform into rutile.^[^ ^99^ ^]^

Several MXene members have excellent conductive properties; for example, the conductivity of Ti_3_C_2_T*_x_* MXene is as high as 2.0 × 10^4^ S cm^−1^, along with high mobility (1 cm^2^ V^−1^ s^−1^) and a high charge carrier density of 3.8 × 10^22^ cm^−3^.^[^ ^100^ ^]^ A number of theoretical and experimental studies have confirmed that bare MXene species, such as Ti*_n_*_+1_X*_n_*, exhibit metallic behavior, and an increase in “*n*” value causes the metallic properties to weaken as additional Ti—X bonds are formed; for example, the metallic nature of Ti*_n_*_+1_N*_n_* T*_x_* (titanium nitride) is stronger than that of Ti*_n_*_+1_C*_n_* T*_x_*, because the N atom possesses one more electron than the C atom.^[^ ^96^, ^100^ ^]^ Further, the metallic properties of MXene can be altered by changing the termination functionalities, and replacing M in the structure.^[^ ^93^ ^]^ Most MXenes are highly conductive, and even superconductive; for example, Nb_2_CT*_x_* (T*_x_* = Se, S, NH), and Mo_2_C exhibit superconductivity.^[^ ^101^, ^102^ ^]^ Talapin et al. reported that Ti_2_CT*_x_* and Ti_3_C_2_T*_x_* MXene decorated with telluride (Te^2−^) ligands exhibited distinctive structural and electronic properties. With Te^2−^ ligand termination, an in‐plane lattice expansion of >18% was observed, which was much higher than that of the pristine titanium carbide lattice.^[^ ^102^ ^]^ They also compared Nb_2_CT*_x_* MXene functionalized with Se, S, and NH, and OH, F, and O terminal groups of MXenes, and the results showed that the conductivity and temperature resistivity are directly influenced by the composition and structure of MXene. The unattainable surface engineering of MXene is expected to influence nearly every property of 2D MXenes, including electronic transport. Ren et al. reported the fabrication of layers of *α*‐Mo_2_C crystals with a thickness of a few nanometers and ≈100 µm in size by chemical vapor deposition (CVD).^[^ ^103^ ^]^ These *α*‐Mo_2_C crystals exhibited thickness‐dependent superconducting transitions that are consistent with Berezinskii–Kosterlitz–Thouless behavior, and revealed strong anisotropy with magnetic field orientation. Moreover, the superconductivity is strongly dependent on the crystal thickness.

In addition to conductive behavior, a few MXenes with suitable surface terminations are expected to exhibit semiconductor‐like behavior. For example, Kawazoe et al. predicted the semiconducting properties of Sc_2_C, Ti_2_C, Zr_2_C, and Hf_2_C MXenes.^[^ ^93^ ^]^ Later research showed that Mo_2_CT*_x_* exhibits semiconductor‐like behavior.^[^ ^104^ ^]^ Most recently, *o*‐MXene was discovered to exhibit semiconductor‐like behavior; for example, Mo_2_TiC_2_T*_x_ o*‐MXene, where the outer Ti layers of Ti_3_C_2_T*_x_* are substituted by Mo, which converts the metallic behavior of Ti_3_C_2_T*_x_* into semiconductor‐like behavior.^[^ ^104^, ^105^ ^]^ The replacement of M has also reportedly influenced the magnetic properties of MXenes. For example, Ti_3_C_2_T*_x_* has antiferromagnetic characteristics; however, the replacement of the outer and middle Ti layers in Ti_3_C_2_T*_x_* with Cr and Mn to produce Cr_2_TiC_2_T*_x_* and TiMn_2_C_2_T*_x_ o*‐MXene, respectively, results in ferromagnetic characteristics.^[^ ^106^, ^107^ ^]^ The superconductive, conductive, or semiconductive‐like characteristics of MXene are entirely dependent upon the surface terminations. Without surface terminations, all MXenes are expected to behave as metallic conductors, where the transition metals are the charge carriers.^[^ ^101^, ^108^, ^109^ ^]^ Unlike conventional metals, in MXenes, DOS and Fermi level changes occur as a result of the surface terminations; thus, bandgap opening, variations in the WFs, and changes in the appearance of semiconductor characteristics are predicted to appear in certain MXenes other than Ti_3_C_2_T*_x_*.^[^ ^86^ ^]^ As discussed earlier, the superconducting transition of Nb_2_CT*_x_* MXene occurs because of its surface termination with Se, S, and NH terminal groups. However, Nb_2_CT*_x_* decorated with O‐termination did not exhibit superconductive behavior. Furthermore, the intercalation of 2D multilayer MXene with organic or inorganic cations during the layer separation process also influences the electronic properties of MXene. For example, the presence of TBA^+^ in the interlayers of MXene could hamper interlayer electron bouncing; as a result, the conductivity of the MXene films decreased.^[^ ^86^, ^87^ ^]^ In the case of inorganic alkali cations, intercalation tends to maintain a smaller interlayer distance and higher conductivity.^[^ ^110^ ^]^

## 3 Thin‐Film and Emerging PV Cells

As mentioned previously, PV cells have emerged as a promising renewable energy source to meet an immensely increasing energy demand. To date, PV cells based on various materials that include inorganics,^[^ ^111^ ^]^ organics,^[^ ^8^ ^]^ the dye‐sensitized,^[^ ^112^ ^]^ and most recently a discovered perovskite,^[^ ^9^ ^]^ have been utilized for light energy harvesting. Amorphous and crystalline silicon solar cells have dominated the other PV technologies because of their well‐matched spectral response with solar irradiation. Since the development of the first inorganic silicon‐based PVs in 1941, an excellent efficiency of beyond 30% for the crystalline silicon PVs has been achieved under standard AM 1.5 G solar illumination conditions (luminescence of 100 mW cm^−2^), due to advancements in device engineering and evolution in fabrication methodologies.^[^ ^113^ ^]^

Though the silicon‐based PVs share the major portion of the PV market, the emerging PVs based on organic, dye, and perovskite materials have gained immense attention due to several advantages that include low cost, solution processability, high mechanical flexibility, easy processing methods, high throughput, and particularly low energy payback times. Since the first report on OPVs in 1981, the efficiency of OPVs was increased from 1% to ≈18% under standard solar illumination conditions.^[^ ^114^ ^]^ The high efficiency of OPVs is attributed to the effective light energy harvesting abilities of the photoactive layer, due to high absorption coefficients, tunable energy levels, and improved charge transport dynamics inside the devices. Similarly, dye‐sensitized solar cells reached an efficiency of over 14% under solar conditions, starting from an efficiency of 7.9% in the DSSCs first developed by Michael Grätzel in 1991.^[^ ^115^ ^]^ The performance of DSSCs has been improved by adopting various design strategies, such as the incorporation of bulky groups in dyes to reduce aggregation, developing long alkyl groups to reduce charge recombination, and extending the *π*‐conjugation of spacers to enhance dye absorption. Moreover, since the last decade, research on PVs has observed a surge with the discovery of perovskite‐based PVs. The perovskite photovoltaic cells (PPVs) have achieved an efficiency of over 25% that is comparable to widely used silicon PVs.^[^ ^116^ ^]^ In addition, the low cost, roll‐to‐roll production methods, and rapid development of PPVs display the undoubtedly great potential of perovskite materials in the PV market. Above all, the excellent photovoltaic performance of emerging PV technologies under indoor light illumination overshadowed the silicon PVs that provided one step forward towards the commercialization of PVs for indoor microelectronic devices.

## 4 Role of MXenes in PV Cells

### 4.1 Additive Engineering

Additive engineering has been demonstrated to be a crucial factor to improve the efficiency of PV cells. Additives in the absorbing or charge transport layers (CTLs) of PV cells perform various functions, such as: I) improving the crystallinity of perovskite materials; II) facilitating tuning of the WFs by modifying the electronic structures; III) boosting the charge transfer; and IV) providing fine morphological control of photoactive layers in solution‐processed PV cells. In this section, we discuss the impact of MXenes as additives on the performance of PV cells.

**Figure 2:**
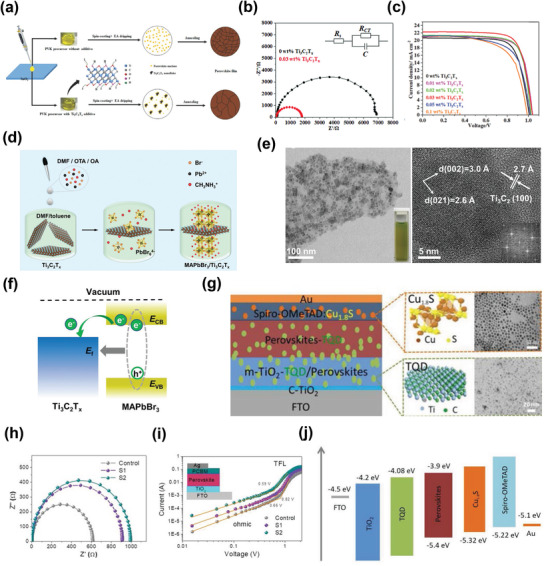
a) Proposed nucleation and growth route of perovskite film with and without Ti_3_C_2_T*_x_* additive. b) Nyquist plots of 0 and 0.03 wt% Ti_3_C_2_T*_x_* additive‐based device measured by EIS under dark with a bias of 0.7 V. c) The *J*–*V* curves of PSCs with different amounts of Ti_3_C_2_T*_x_*. Reproduced with permission.^[^ ^69^ ^]^ Copyright 2018, Wiley‐VCH. d) Schematic of the synthesis process of MAPbBr_3_/Ti_3_C_2_T*_x_* heterostructures. e) TEM images of the MAPbBr_3_/Ti_3_C_2_T*_x_*‐1 heterostructures: the deposition of MAPbBr_3_ nanocrystals (NCs) on the surface of Ti_3_C_2_T*_x_* (left), and the diffraction pattern of the lattice area of MAPbBr_3_ NCs (right). f) Schematics of the band alignment and proposed energy transfer process between MAPbBr_3_ and Ti_3_C_2_T*_x_* nanosheets. Reproduced with permission.^[^ ^118^ ^]^ Copyright 2020, Wiley‐VCH). g) Architecture of the perovskite solar cells (left), with schematic structures and TEM images of Cu_1.8_S and Ti_3_C_2_ QDs (right). h) Nyquist plots measured by EIS analysis. i) Double logarithmic *J*–*V* characteristics in electron‐only devices with the FTO/TiO_2_/perovskite/PCBM/Ag structure for the control, S1, and S2 devices. j) Energy diagram of each layer in PSCs. Reproduced with permission.^[^ ^121^ ^]^ Copyright 2020, Wiley‐VCH.

The exploration of the incorporation of MXenes in PV cells was initiated by Ma et al. in 2018, where they used Ti_3_C_2_T*_x_* as an additive in the absorbing layer of MAPbI_3_‐based perovskite cells to improve the photovoltaic performance.^[^ ^69^ ^]^ They observed that the incorporation of Ti_3_C_2_T*_x_* significantly increased the grain size of the CH_3_NH_3_PbI_3_ film owing to the slow nucleation and growth rate (**Figure** **2**a). The large crystal size enhanced the light absorption ability of the perovskite layer. Additionally, the high electrical conductivity and mobility of Ti_3_C_2_T*_x_* were found to be advantageous for boosting the charge‐carrier transfer inside the device. The excellent charge transfer characteristics of the devices with the Ti_3_C_2_T*_x_* additive were validated by electrochemical impedance spectroscopy (EIS) analysis, as shown in Figure 2b.^[^ ^117^ ^]^ As a result, the power conversion efficiency of the Ti_3_C_2_T*_x_*‐employed MAPbI_3_‐based solar cells was enhanced from 15.5% to 17.4% at an optimal 0.03 wt% of Ti_3_C_2_T*_x_* additive. Figure 2c displays the current density–voltage (*J*–*V*) curves of the polymer solar cells (PSCs) with different amounts of Ti_3_C_2_T*_x_*. This study paved the way for exploring various variants of MXenes in PV technology. Similarly, Xing et al. deposited perovskite nanocrystals on few‐layer Ti_3_C_2_T*_x_* nanosheets to develop perovskite/MXene interfaces by an in situ solution growth process, aiming to improve the energy transfer characteristics.^[^ ^118^ ^]^ Figure 2d shows a schematic of the synthetic procedure of the MAPbBr_3_/Ti_3_C_2_T*_x_* heterostructures. The TEM images of the MAPbBr_3_/Ti_3_C_2_T*_x_*‐1 heterostructures show the deposition of MAPbBr_3_ nanocrystals (NCs) on the surface of Ti_3_C_2_T*_x_* (Figure 2e, left) and the diffraction pattern of the lattice area of MAPbBr_3_ NCs (Figure 2e, right). Extensive investigations revealed that the energy transfer between perovskite and Ti_3_C_2_T*_x_* heterostructures was enhanced by increasing the essential composition of Ti_3_C_2_T*_x_*, as shown by the schematic of band alignment (Figure 2f). The findings in this study provide guidelines for the use of perovskite/MXene heterostructures that are useful for realizing efficient optoelectronic devices. Moreover, Di Carlo et al. examined the influence of Ti_3_C_2_T*_x_* on the charge‐transfer properties at the perovskite/TiO_2_ interface.^[^ ^65^ ^]^ They added Ti_3_C_2_T*_x_* to MAPbI_3_ films to optimize the energy‐level alignment at the MAPbI_3_/TiO_2_ electron transport layer (ETL), and thereby observed a shift in the WF in the range 4.72–4.37 eV, without affecting other electronic properties. They showed that Ti_3_C_2_T*_x_* can effectively improve the charge transfer by adjusting the WF between the halide perovskite film and the TiO_2_ ETL. The inimitable WF flexibility of MXenes shown in this study may encourage novel designs of perovskite‐based electronic devices in the future.^[^ ^118^, ^119^ ^]^ Recently, Song et al. utilized ultrathin Ti_3_C_2_T*_x_* quantum dots (TQDs) in perovskite absorber films and interfacial layers (ETL and hole transport layer [HTL]) to realize efficient perovskite‐based PV cells.^[^ ^121^ ^]^ Figure 2g (left) shows the architecture of perovskite solar cells, while Figure 2g (right) shows the schematic structure and TEM images of Cu_1.8_S and Ti_3_C_2_ QDs. They examined the charge transport dynamics by EIS analysis, as shown in the Nyquist plots (Figure 2h). TQDs decrease the intrinsic defects and grain size boundaries by passivating the charge carrier recombination zones, thus accelerating the charge carrier transport properties, as evident in the *J*–*V* curves of electron‐only devices (Figure 2i). Furthermore, the inclusion of TQDs in the perovskite absorbing layer improves the conductivity, band alignment at the interface (Figure 2j), and crystallinity of the perovskite films.

### 4.2 Interfacial Engineering

The choice of appropriate interfacial materials, so‐called CTLs, is exceptionally critical for securing high‐performance PV cells.^[^ ^121^, ^122^, ^123^ ^]^ In both conventional and inverted structures, a photoactive layer is inserted between the indium tin oxide (ITO) electrode and the metal electrode. CTLs labeled as either the HTL or ETL, according to their functionalities, are employed to adjust the photoactive layer/electrode interfaces. These CTLs perform various important functions, such as tuning the polarity of the electrodes (hole‐collecting or electron‐collecting), adjusting the energy barriers between the photoactive layer and the electrodes for efficient charge carrier transportation, preventing oxygen and moisture from penetrating the photoactive layer, and finally choosing one type of charge carrier and reducing charge carrier recombination.^[^ ^124^, ^125^, ^126^, ^127^, ^128^, ^129^ ^]^ The excellent conductivity, high optical transmittance, tunable WF, and outstanding mobility of MXenes make them promising candidates for interfacial materials. Related studies of MXene as interfacial layers in organic photovoltaics (OPVs), perovskites, and silicon solar cells are subsequently discussed.

**Figure 3:**
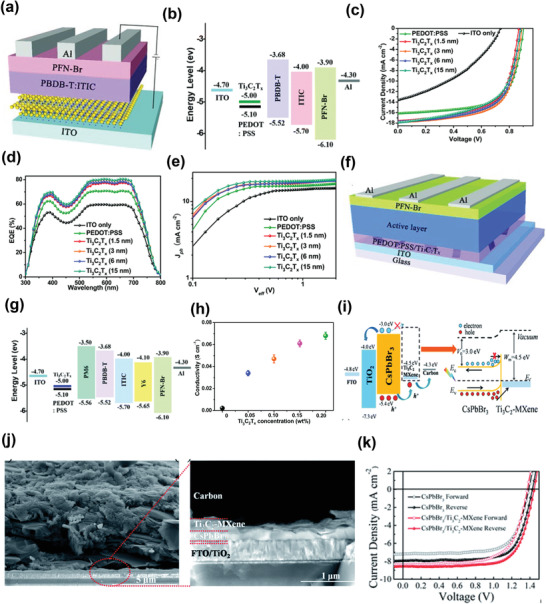
a) Schematic of the PSC architecture. b) Energy band diagrams of the used materials. c) *J*–*V* characteristics of devices with different HTLs under illumination. d) The corresponding EQE curves. e) *J*_ph_–*V*_eff_ curves of devices with different HTLs. Reproduced with permission.^[^ ^130^ ^]^ Copyright 2019, Royal Society of Chemistry). f) Schematic of the devices. g) Energy level diagram of the materials. h) Conductivities of the PEDOT:PSS and PEDOT:PSS/Ti_3_C_2_T*_x_* films on bare glass. Reproduced with permission.^[^ ^131^ ^]^ Copyright 2020, Royal Society of Chemistry. i) Energy bandgap of the device under illumination and carrier transport mechanism of the device at the interface. j) Cross‐sectional SEM image of the device: low magnification (left) and high magnification (right). k) *J*–*V* curves of PSCs. Reproduced with permission.^[^ ^72^ ^]^ Copyright 2019, Royal Society of Chemistry.

In 2019, Huang et al. employed Ti_3_C_2_T*_x_* nanosheets as the HTL material in PSCs, as shown in the schematic of PSCs of **Figure** **3**a.^[^ ^130^ ^]^ The high conductivity, matched energy band alignments (Figure 3b), and improved interface contacts of Ti_3_C_2_T*_x_* HTL in PSCs based on (poly[(2,6‐(4,8‐bis(5‐(2‐ethylhexyl)thiophen‐2‐yl)benzo[1,2‐b:4,5‐b0]‐dithiophene)‐co‐(1,3‐di(5‐thiophen‐2‐yl)‐5,7‐bis(2‐ethylhexyl)benzo[10,20‐c:4,5‐c0]dithiophene‐4,8‐dione))]) (PBDB‐T):3,9‐bis(2‐methylene‐(3‐(1,1‐dicyanomethylene)‐indanone))‐5,5,11,11‐tetrakis(4‐hexylphenyl)‐dithieno[2,3‐d:20,30‐d0]‐s‐indaceno[1,2‐b:5,6‐b0]dithiophene) (ITIC) photoactive layer yielded improved hole transport and collection characteristics. The Ti_3_C_2_T*_x_*‐incorporated devices demonstrated an excellent PCE of 10.5%, whereas that of indium tin oxide (ITO)‐based devices was only ≈4.6%. Figure 3c shows the *J*–*V* curves of PSCs employing different HTLs, while Figure 3d gives the corresponding EQE spectra. Notably, the Ti_3_C_2_T*_x_*‐employed devices also surpassed the commonly used poly(3,4‐ethylenedioxythiophene) polystyrene sulfonate (PEDOT:PSS)‐based device (10.5% versus 10.1%). The charge carrier transport and extraction properties of the PSCs with PEDOT:PSS and Ti_3_C_2_T*_x_* HTLs were measured by plotting photogenerated current density as a function of light intensity (Figure 3e). Additionally, the long‐lasting stability of Ti_3_C_2_T*_x_*‐based devices in an ambient environment compared to PEDOT:PSS‐based devices demonstrate the great potential of Ti_3_C_2_T*_x_* as an interfacial material.

In the following year, Huang et al. reported a nanostructured composite of PEDOT:PSS/Ti_3_C_2_T*_x_* as an HTL for PBDB‐T:ITIC‐based PSCs (Figure 3f).^[^ ^131^ ^]^ They achieved a relatively high PCE of 11.02% with the PEDOT:PSS/Ti_3_C_2_T*_x_* composite, compared with 9.72% of the pristine PEDOT:PSS‐based device. An improvement in charge transfer was observed between the PEDOT nanocrystals when Ti_3_C_2_T*_x_* nanoflakes were included, because of the closer energy alignment (Figure 3g). In addition, the metallic conductivity of the PEDOT:PSS/Ti_3_C_2_T*_x_* composite was substantially enhanced by the conformational evolution of PEDOT from the coil to the expanded coil structure. Figure 3h shows the conductivities of the composite with a varied ratio of Ti_3_C_2_T*_x_*. Moreover, the PCE was enhanced from 13.1% to 14.5% for the pristine PEDOT:PSS and PEDOT:PSS‐modified in PM6:Y6‐employed PSCs, respectively. The excellent performance of the PEDOT:PSS/Ti_3_C_2_T*_x_* composite devices offers promising prospects for use in optoelectronic applications.^[^ ^131^, ^132^, ^133^ ^]^

In 2019, Jiang et al. introduced Ti_3_C_2_T*_x_* nanosheets as the HTL in all‐inorganic CsPbBr_3_ solar cells.^[^ ^72^ ^]^ The improved energy level alignment between the photoactive layer and the carbon electrode resulted in decreased charge carrier recombination at the interface, owing to the fast hole extraction and electron blocking ability of the Ti_3_C_2_T*_x_* nanosheets (Figure 3i). Moreover, the surface defect densities in the absorbing photoactive layer were lowered because of the passivation of CsPbBr_3_ grains by the functional groups in Ti_3_C_2_T*_x_*, as shown by cross‐sectional images of the SEM in Figure 3j, thereby improving the quality of the perovskite film. As a result, the devices based on Ti_3_C_2_T*_x_* HTLs exhibited a PCE of 9.01%, along with high current density (Figure 3k) and improved thermal stability. The findings of this study pave the way toward realizing high‐performance and stable all‐inorganic perovskite solar cells by including a unique interlayer. Compared to HTLs, few studies have been conducted on ETLs that employ MXenes.

**Figure 4:**
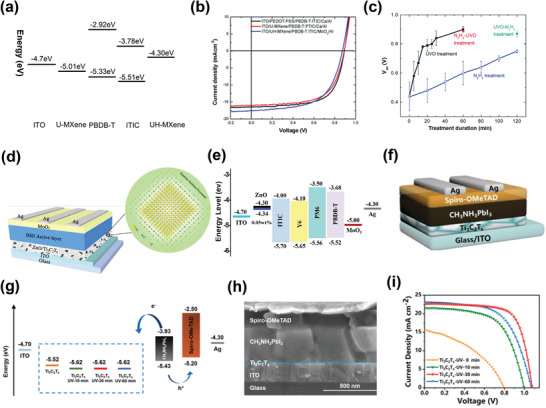
a) Schematic of the energy levels of different materials; the WFs of the U‐MXene and UH‐MXene were determined by the KPFM test. U‐MXene (UVO‐treated MXene) is used for hole collection in the normal OSC, and UH‐MXene (N_2_H_4_‐treated MXene) is used for electron collection in the inverted OSC. b) *J*–*V* curves of PBDB‐T:ITIC organic solar cells (OSCs) with the normal and inverted architecture. c) Variation in *V*_OC_ with the treatment duration. Devices exposed to UVO and N_2_H_4_‐UVO exhibited conventional behavior, as opposed to those treated with N_2_H_4_ and UVO‐N_2_H_4_. Reproduced with permission.^[^ ^66^ ^]^ Copyright 2019, Royal Society of Chemistry. d) Schematic of the inverted polymer solar cells (IPSCs) configuration. e) Band diagram of the materials used in IPSCs. Reproduced with permission.^[^ ^134^ ^]^ Copyright 2021, Elsevier. f) Device architecture of the ITO/ETL/CH_3_NH_3_PbI_3_/Spiro‐OMeTAD/Ag‐based on Ti_3_C_2_T*_x_* with/without UV‐ozone treatment as ETL. g) Schematic energy level of each layer. h) Cross‐sectional SEM image of the PSC. i) *J*–*V* curves of PSCs based on Ti_3_C_2_T*_x_* with UV‐ozone treatment for different times as ETL under simulated illumination (AM 1.5 G). Reproduced with permission.^[^ ^135^ ^]^ Copyright 2019, Wiley‐VCH.

In 2019, Ouyang et al. investigated the role of Ti_3_C_2_T*_x_* as an ETL and HTL in PBDB‐T:ITIC‐based OPVs.^[^ ^66^ ^]^ They revealed that the WF of MXene can be modulated, that is, increased or decreased (from 4.95 to 4.08 eV) using ultraviolet‐ozone (UVO) treatment labeled as (u‐MXene), or N_2_H_4_ treatment labeled as (UH‐MXene), respectively, as shown in the energy levels diagram of materials (**Figure** **4**a). The UVO and N_2_H_4_ treatments resulted in the oxidation and reduction of C in Ti_3_C_2_T*_x_*, respectively. They achieved efficiencies of 9.06% and 9.02% with Ti_3_C_2_T*_x_*‐based ETLs and HTLs, respectively. The PCEs of the Ti_3_C_2_T*_x_*‐based CTLs were comparable to those (9.67%) of the OPVs employing PEDOT:PSS and Ca as HTL and ELT, respectively. Figure 4b provides the *J*–*V* curves of the devices with inverted and normal geometry. They also analyzed the influence of the duration of the treatment on the photovoltaic performance of OPVs, as shown in Figure 4c. Because the open‐circuit voltage (*V*_OC_) depends on the energy levels between the photoactive layer and the electrode, the evolution of the *V*_OC_ values was consistent with the variation in the WF of MXenes.

More recently, Yu et al. developed a novel Zinc oxide (ZnO)/ Ti_3_C_2_T*_x_* composite film for inverted PBDB‐T:ITIC‐based OPVs (Figure 4d).^[^ ^134^ ^]^ The proposed hybrid composite film, working as an ETL, exhibited excellent optoelectrical characteristics. The inclusion of Ti_3_C_2_T*_x_* nanosheets in the sol–gel ZnO precursor resulted in Zn—O—Ti bonding on the surface of ZnO, thereby leading to charge transport pathways inside the ZnO nanocrystals. Thus, PBDB‐T:ITIC‐incorporated OPVs with ZnO/Ti_3_C_2_T*_x_* ETLs demonstrated a PCE of 12.2%, which was 15% higher than that (10.5%) of the OPVs employing pure ZnO ETL. The universal applicability of the ZnO/Ti_3_C_2_T*_x_* ETLs was further validated by utilizing them in PM6:Y6‐based OPVs, where they exhibited a PCE of 16.51%, which was slightly higher (14.9%) than the reference OPVs. The modified ZnO layer contributed to the simultaneous improvement in the *J*_SC_ and FF values, owing to the increased charge transfer paths in the ZnO/Ti_3_C_2_T*_x_* films. Figure 4e shows the energy band diagram of materials. In 2019, Miyasaka et al. used Ti_3_C_2_T*_x_* as an ETL in low‐temperature processed MAPbI_3_‐based solar cells (Figure 4f).^[^ ^135^ ^]^ The Fermi energy level of the layer was varied between −5.52 and −5.62 eV with exposure to UVO for 30 min, as depicted in Figure 4g. The enhanced interfacial properties between the perovskite and Ti_3_C_2_T*_x_* layers were attributed to the formation of additional oxide‐like Ti—O bonds on the Ti_3_C_2_T*_x_* surface. Figure 4h shows the cross‐sectional image of PSC. The UVO treatment of the Ti_3_C_2_T*_x_* layer resulted in the improvement of electron transfer and reduction of charge recombination at the perovskite/ETL interface. Consequently, an excellent PCE of 17.1% was achieved with the UVO‐treated devices, in comparison to the devices without UVO treatment (5.00%), with significantly improved current density (Figure 4i).

**Figure 5:**
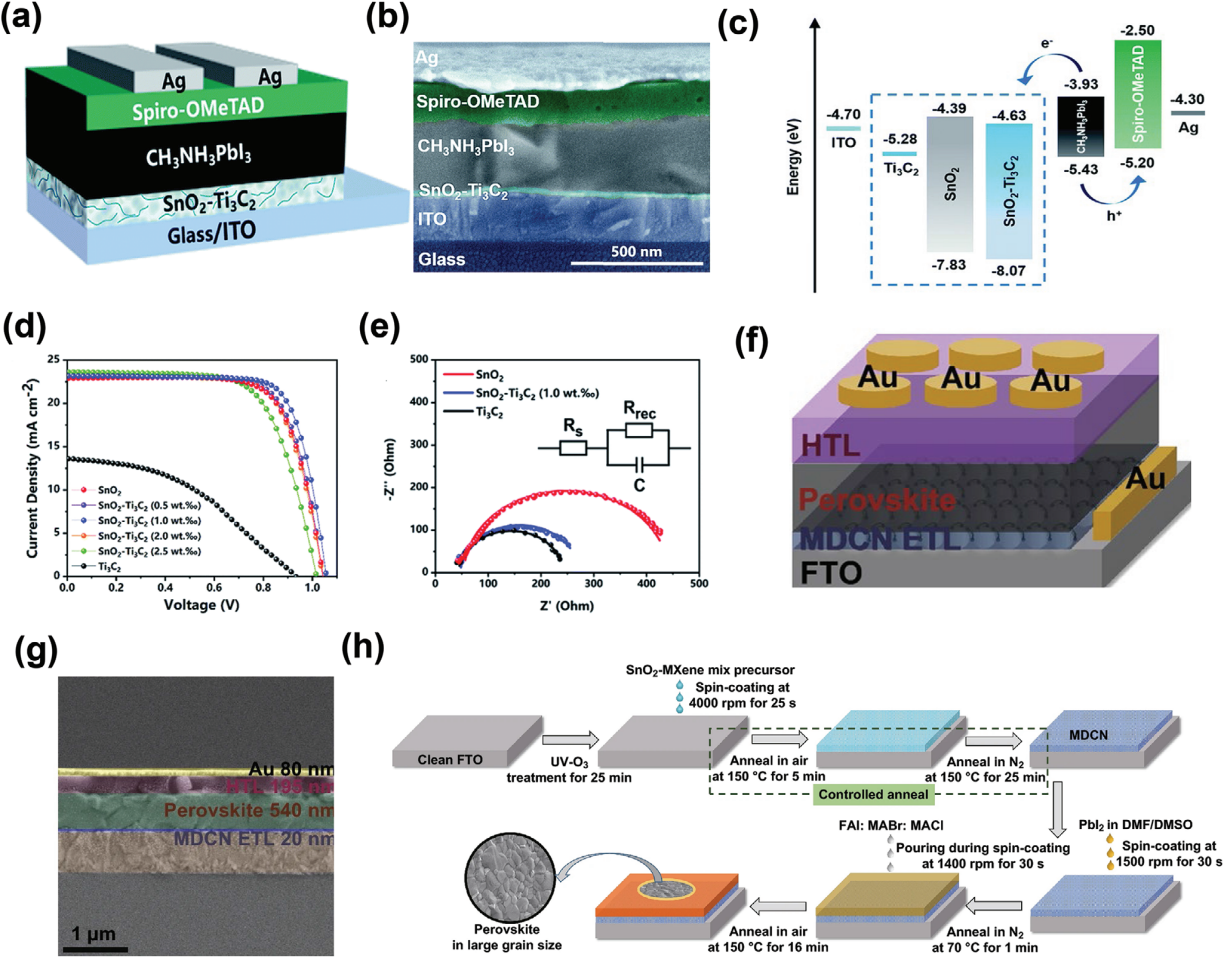
a) Device architecture of the ITO/ETL/CH_3_NH_3_PbI = /Spiro‐OMeTAD/Ag‐based on representative SnO_2_—Ti_3_C_2_ as the ETL. b) Cross‐sectional SEM image of the PSC device and c) schematic energy level diagram of each layer. d) *J*–*V* curves of PSCs based on SnO_2_, SnO_2_—Ti_3_C_2_ (prepared with different concentrations of Ti_3_C_2_), and Ti_3_C_2_ as ETLs under AM 1.5 G simulated illumination. e) Nyquist plots of the PSCs with SnO_2_, SnO_2_—Ti_3_C_2_ (1.0 wt%), or Ti_3_C_2_ as ETLs under one sun illumination (Reproduced with permission.^[^ ^71^ ^]^ Copyright 2019, Royal Society of Chemistry). f) The device structure of a PSC using MDCN as ETL. g) Cross‐sectional SEM image of a complete device with MDCN‐0.02 ETL. h) Fabrication process of the perovskite layer with MDCN ETL. Reproduced with permission.^[^ ^136^ ^]^ Copyright 2020, Springer Nature.

In the same year, Miyasaka et al. extended their investigations on the interfacial properties of the Ti_3_C_2_T*_x_* layer in perovskite solar cells by introducing Ti_3_C_2_/SnO_2_ nanocomposites with various concentrations of Ti_3_C_2_.^[^ ^71^ ^]^ **Figure** **5**a shows a schematic of the perovskite solar cells, while Figure 5b shows a cross‐sectional SEM image of the device. The PCE of the PSCs was enhanced from 17.2% to 18.3% at an optimal concentration of 1.0 wt%. The improved performance of the Ti_3_C_2_/SnO_2_ nanocomposite‐based solar cells was ascribed to the high mobility, suitable energy level alignments (Figure 5c), improved charge transfer paths, and low charge transfer resistance at the interface. Figure 5d displays the improvement in the current density with the nanocomposites, whereas Figure 5e shows the Nyquist plot using EIS analysis, which figures confirmed the efficient charge carrier characteristics. The utilization of low‐temperature solution‐processed, and more affordable ETLs in this study suggested versatile applications for flexible optoelectronic devices. Another in‐depth study on interfacial layers incorporating SnO_2_, TiO_2_, and MXene composite layers labeled as multidimensional conductive networks (MDCNs) was conducted by Liang et al. in 2020.^[^ ^136^ ^]^ They introduced anatase TiO_2_ quantum dots (QDs) into a Ti_3_C_2_T*_x_*/SnO_2_ heterojunction structure, as shown in the device structure of PSCs (Figure 5f). The suitable band alignment of the MDCN ETL with the perovskite absorbing layer resulted in a high PCE of 19.14%. In contrast, the devices with the SnO_2_ ETL exhibited a PCE of 16.83%. Figure 5g shows the cross‐sectional SEM image of the PSC with MDCN ETL. The conductive bond bridges of MXene contributed to an improvement in the conductivity of the ETL. Additionally, the optical transmittance, crystallinity of the upper perovskite absorbing layer, and the ETL/perovskite interface were improved. Figure 5h represents the complete fabrication process of the perovskite absorbing layer with MDCN ETL.

**Figure 6:**
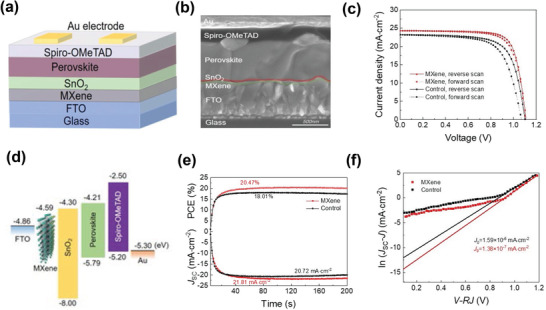
a) Device configuration used in this study. b) Cross‐sectional SEM image of the entire device. c) Standard *J*–*V* characteristics of control and MXene‐modified PSCs under AM 1.5 G 100 mW cm^−2^ illumination. d) Flat‐band energy level diagram of the MXene‐modified device. e) Steady‐state efficiencies and *J*_SC_ of control and MXene‐modified PSCs at their maximum power points. f) Dark saturated current density *J*_0_ of control and MXene‐modified PSCs derived from dark *J*–*V* curves. Reproduced with permission.^[^ ^137^ ^]^ Copyright 2020, American Chemical Society.

In 2019, Di Carlo et al. also introduced MXene in a TiO_2_ ETL to tune the WF at the MAPbI3/ETL interface.^[^ ^65^ ^]^ The Ti_3_C_2_T*_x_*‐engineered interfaces yielded a PCE in excess of 20%, which was almost 26% higher than that of the reference devices without MXene. In addition to the improvement in efficiency, the devices with ETLs exhibited low hysteresis in the *J*–*V* characteristics. Interface engineering with MXene is an important design criterion for achieving efficient charge transfer characteristics and enhanced device performance. Recently, Zhao et al. fabricated planar PSCs with a SnO_2_ ETL modified with 2D titanium‐carbide MXenes, as shown in the schematic of PSCs (**Figure** **6**a).^[^ ^137^ ^]^ Figure 6b shows a cross‐sectional image of the PSCs. Figure 6c shows the *J*–*V* curves of control and MXene‐based PSCs under 1 sun illumination. The introduction of Ti_3_C_2_T*_x_* between the SnO_2_ ETL and bottom F‐doped SnO_2_ electrode provided suitable band alignment between the ETL and FTO, as displayed in Figure 6d, and preferred electron hybridization, thus leading to lower recombination losses, excellent homogeneity, and improved electron mobility between the ETL and FTO. Steady‐state efficiencies and the *J*_SC_ of the MXene‐modified PSCs were found slightly higher than for the control PSCs (Figure 6e). In addition, the MXene‐containing SnO_2_ ETL resulted in smooth and hydrophobic surfaces, providing a promising growth platform for high‐quality perovskite layers. As a result, the synergistic influence of MXene in the SnO_2_ ETL resulted in negligible hysteresis losses and ultralow saturated current density as shown in Figure 6f, leading to an excellent PCE of 20.6%, which was higher than that of the control device without MXene (PCE of control device: 19.0%). The comprehensive study of MXene interfacial engineering in this work provides alternative design strategies to develop efficient PSCs.

### 4.3 Transparent Conductive Electrodes

Developing highly transparent and conductive TCEs is a crucial factor for achieving high efficiency in PV cells.^[^ ^137^, ^138^, ^139^, ^140^, ^141^ ^]^ The high transparency of the TCEs allows more photons to be harvested, thus leading to increased absorption. The high cost of carbon‐based electrodes limits their application in PV technology. Alternatively, the excellent electrical conductivity, high optical transmittance, low cost, and suitable band alignment of MXenes make them ideal candidates for TCEs. The following section discusses the role and applications of MXenes as TCEs in different types of PV cells.

**Figure 7:**
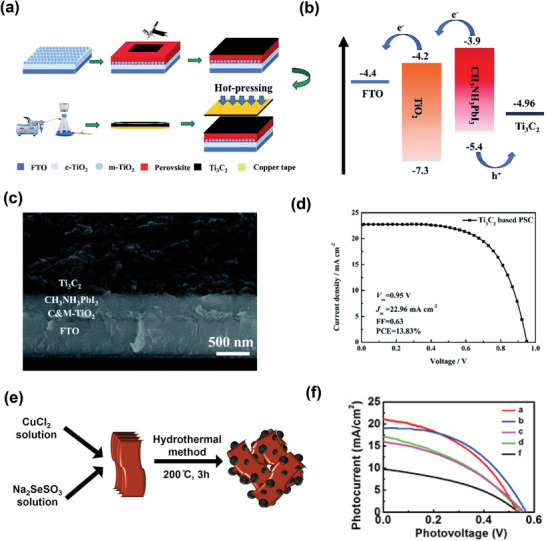
a) Schematic of the fabrication process of the Ti_3_C_2_ electrode by the hot‐pressing method. b) Energy level diagram of the PSCs, and c) cross‐sectional SEM image of the PSC based on Ti_3_C_2_ electrode. d) *J*–*V* curves of the champion device based on the Ti_3_C_2_ electrode (Reproduced with permission.^[^ ^91^ ^]^ Copyright 2019, Royal Society of Chemistry). e) Schematic of the synthesis process of the CuSe/Ti_3_C_2_ composite and f) *J*–*V* characterization. Reproduced with permission.^[^ ^142^ ^]^ Copyright 2019, Elsevier.

In 2019, Ma et al. utilized MXene (Ti_3_C_2_) as the back electrode in PSCs for the first time.^[^ ^91^ ^]^ They used the hot‐pressing technique to create a seamless interfacial contact between the MXene electrode and the perovskite absorbing layer. **Figure** **7**a represents the complete fabrication process of the Ti_3_C_2_ electrode. The suitable energy level alignment between the MAPbI_3_ absorbing layer and Ti_3_C_2_ back electrode (Figure 7b) and the higher conductivity improve the charge carrier dynamics, thus leading to a champion PCE of 13.83%, which was 27% higher than that (10.87%) of the carbon electrode‐based PSCs. Figure 7c depicts the cross‐sectional SEM image of the complete device, while Figure 7d shows the *J*–*V* curve of the champion device with Ti_3_C_2_ electrode. This study suggests a unique application of MXene as a metal‐free electrode for PSCs. In the same year, Xie et al. devised a hybrid structure based on CuSe nanoparticles and 2D MXene (Ti_3_C_2_) nanosheets.^[^ ^142^ ^]^ The hybrid structure deposited on a graphite sheet through screen‐printing was first applied as a counter electrode (CE) in quantum‐dot dye‐sensitized solar cells (QDSSCs). Figure 7e illustrates the synthetic process of hybrid CuSe/Ti_3_C_2_ nanosheets. In comparison to the pure Ti_3_C_2_ and CuSe CEs, the hybrid CE improves the electrical conductivity and surface area, thus facilitating charge carrier extraction and the reduction of polysulfide electrolytes by providing more active sites. The devices with CuSe and Ti_3_C_2_ exhibited PCE values of 3.47% and 2.04%, respectively, whereas the PCE of the device with the hybrid CuSe/Ti_3_C_2_ CE increased up to 5.12%. The improvement in the current densities is shown in the *J*–*V* curves (Figure 7f). This study recommends an effective strategy for the design of novel electrodes for DSSCs.

**Figure 8:**
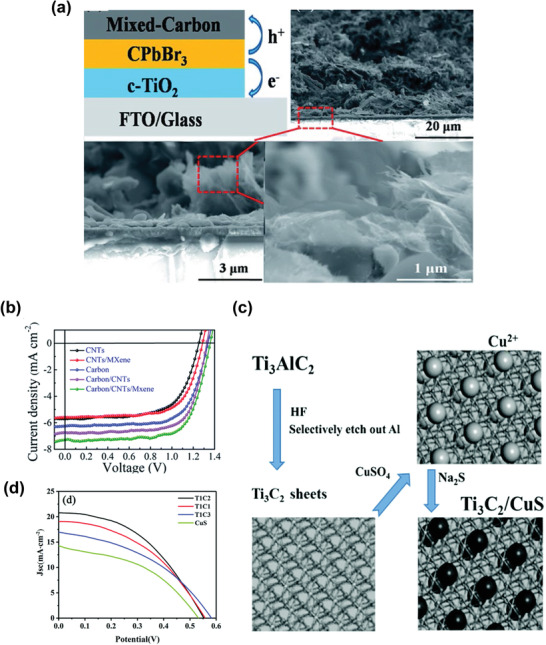
a) Schematic structure of the mixed carbon electrode CsPbBr_3_ solar cell with cross‐sectional SEM images of the devices, and b) *J*–*V* curves of the devices with different types of electrodes. Reproduced with permission.^[^ ^143^ ^]^ Copyright 2020, Royal Society of Chemistry. c) Schematic of the synthetic method for the fabrication of Ti_3_C_2_/CuS using an ion‐exchange method. d) *J*–*V* plots of QDSSCs based on various CEs under simulated solar illumination (AM 1.5 G). Reproduced with permission.^[^ ^144^ ^]^ Copyright 2020, Royal Society of Chemistry.

Subsequently, Jiang et al. introduced a composite electrode based on a mixture of carbon paste, 1D carbon nanotubes (CNTs), and 2D Ti_3_C_2_T*_x_* nanosheets in all‐inorganic PSCs. **Figure** **8**a shows a schematic of the composite electrode‐based PSC with a cross‐sectional SEM image of the entire device.^[^ ^143^ ^]^ The composite electrode proposed in this study provides point‐to‐point contact, which leads to multidimensional charge carrier dynamics through a network structure, thus effectively enhancing the electrical conductivity of the electrode and charge carrier transport. The excellent interfacial properties of the newly designed composite electrode resulted in a notable PCE of 7.09%, due to improvement in the current densities (Figure 8b). The findings of this study demonstrate the potential of MXene‐based electrodes for the large‐scale deployment of PSCs. Similarly, Zhong et al. developed a Ti_3_C_2_/CuS composite as a counter electrode for QDSSCs.^[^ ^144^ ^]^ Figure 8c shows a schematic of the synthetic procedure of the composite electrode, in which the CuS nanoparticles were anchored on Ti_3_C_2_ using the ion‐exchange method at room temperature (RT). The Ti_3_C_2_/CuS composite reduced polysulfide at a faster electrocatalytic rate than that of the pure CuS. The QDSSCs employing the Ti_3_C_2_/CuS electrode yielded a PCE of 5.11%, which was 1.5% higher than that of the device with the CuS counter electrode (3.26%). Figure 8d displays the *J*–*V* curves of the devices. The high conductivity of Ti_3_C_2_ MXene and the excessive catalytically active sites of the CuS nanoparticles contributed to the improvement in the performance of the QDSSCs.

**Figure 9:**
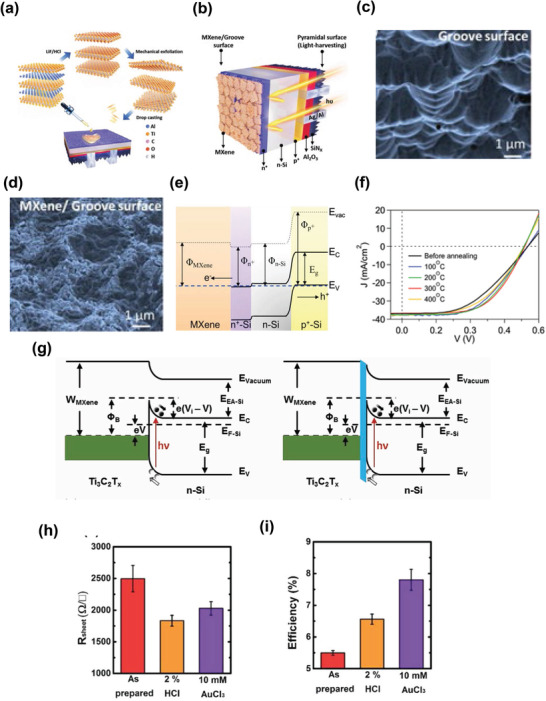
a) Schematic of the synthesis of delaminated Ti_3_C_2_T*_x_* MXene sheets and the fabrication of the Ti_3_C_2_T*_x_* MXene/n+np+‐Si solar cell device by drop‐casting delaminated Ti_3_C_2_T*_x_* MXene on the n+‐Si surface. b) Device architecture of Ti_3_C_2_T*_x_* MXene/n+np+‐Si solar cell. c,d) FE‐SEM images of the p+ side, and Ti_3_C_2_T*_x_* MXene deposited n+side of the n+np+‐Si solar cell. e) Proposed band diagram of the Ti_3_C_2_T*_x_* MXene/n+np+‐Si solar cell based on the calculated WF measurements. f) *J*–*V* characteristics of the Ti_3_C_2_T*_x_* MXene/n+np+‐Si solar cell before and after the rapid thermal annealing (RTA) process from 100 to 400 °C under AM 1.5 illumination (measured in air at a scan rate of 20 mV s^−1^). Reproduced with permission.^[^ ^145^ ^]^ Copyright 2020, Wiley‐VCH. g) Band diagrams of (left) Ti_3_C_2_T*_x_*/Si, and (right) Ti_3_C_2_T*_x_*/SiO_2_/Si, heterojunction. h) Sheet resistance of the devices. i) Efficiency of the devices. Reproduced with permission.^[^ ^90^ ^]^ Copyright 2019, Wiley‐VCH.

Likewise, He et al. used few‐layered 2D Ti_3_C_2_T*_x_* nanosheets as the material for the back electrode in an n+–n–p+ silicon solar cell and secured a PCE of 11.5% under outdoor illumination.^[^ ^145^ ^]^ **Figure** **9**a shows the synthesis procedure of delaminated Ti_3_C_2_T*_x_* sheets and fabrication of the Ti_3_C_2_T*_x_* MXene/n+np+‐Si solar cell device using drop casting delaminated Ti_3_C_2_T*_x_* on the surface of n+‐Si, while Figure 9b shows the device structure. FE‐SEM images of the p+ side (Figure 9c), and Ti_3_C_2_T*_x_* MXene deposited n+side (Figure 9d) of the n+np+‐Si solar cell are also presented. An ohmic junction was developed between MXene and n+‐Si, which assisted with decreasing the contact resistance, extraction of charge carriers from the n+np+‐Si network, and less recombination, thereby resulting in sufficiently improving the *V*_OC_ and *J*_SC_ values. Figure 9e shows the energy diagram of PVs. Additionally, the electrical contact was improved by applying rapid thermal annealing for 30 s, which contributed to boosting the *J*_SC_ (Figure 9f), FF, and consequently PCE values, because of a decrease in sheet and series resistances, and an increase in metallic conductivity. The findings of this study suggest that solution‐processed MXene can be a suitable applicant for fabricating efficient PV systems, and other types of MXene compositions can also be explored.

In the same year, Shapter et al. extended the investigation of the role of MXene as an electrode and hole‐collecting layer in silicon solar cells.^[^ ^90^ ^]^ They deposited Ti_3_C_2_T*_x_* nanosheets on a Si‐heterojunction network, and achieved an initial PCE of ≈5.0%. Figure 9g gives the energy band diagram of the devices with Ti_3_C_2_T*_x_*/Si (left), and Ti_3_C_2_T*_x_*/SiO_2_/Si (right), heterojunction. Furthermore, they applied chemical treatments (HCl and AuCl_3_) to Ti_3_C_2_T*_x_*‐on Si‐based solar cells, and observed a 9% increase in the PCE values. The HCl treatment contributed to the improvement in conductivity owing to the doping effect, whereas the charge transfer dynamics were substantially enhanced by exposure to AuCl_3_. Figure 9h depicts the values of sheet resistances with various chemical treatments. An ultrathin layer of SiO_2_ plays a significant role in reducing the charge carrier recombination, because of the improvement in the junction. They achieved a PCE exceeding 11% after chemical treatment, with excellent reproducibility. Figure 9i summarizes the improvement of PCE values with chemical treatment. The authors anticipated that their study would encourage the research community to explore a versatile class of 2D materials for future applications.

**Figure 10:**
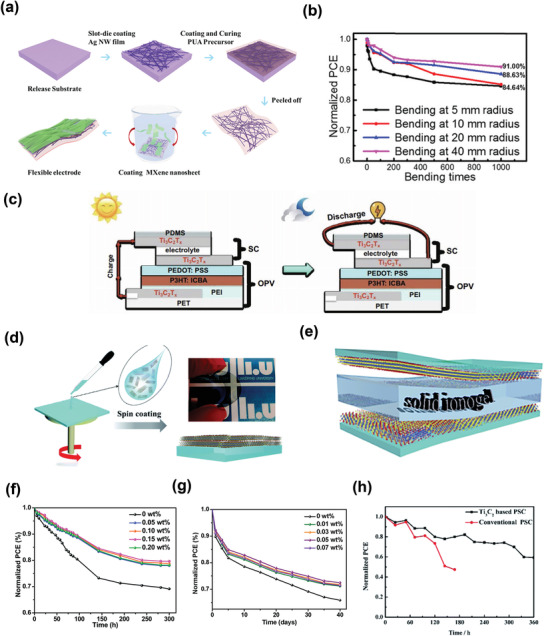
a) Fabrication process of the MXene‐based flexible transparent electrode. b) Normalized PCE of the flexible PSCs with MXene/AgNW‐PUA transparent electrodes at different bending radii as a function of the number of bending cycles. Reproduced with permission.^[^ ^146^ ^]^ Copyright 2019, American Chemical Society. c) Device structure of OPVs integrated with the capacitor. d) Schematic of the preparation of a transparent flexible electrode. e) Schematic of a flexible solid‐state supercapacitor based on spin coating Ti_3_C_2_T*_x_* MXene on PET sheets as electrodes, and solid ionogel as the electrolyte. Reproduced with permission.^[^ ^147^ ^]^ Copyright 2020, Royal Society of Chemistry. f) Stability of devices with different HTLs based on PBDB‐T:ITIC in a N_2_ glove box. Reproduced with permission.^[^ ^131^ ^]^ Copyright 2020, Royal Society of Chemistry). g) Stability of IPSCs based on PBDB‐T:ITIC without encapsulation in the air. Reproduced with permission.^[^ ^134^ ^]^ Copyright 2021, Elsevier. h) Stability tests of the Ti_3_C_2_ electrode‐based PSCs and conventional PSCs in an ambient atmosphere at RT (humidity 30%). Reproduced with permission.^[^ ^91^ ^]^ Copyright 2019, Royal Society of Chemistry.

In 2019, Chen et al. successfully developed transparent, conductive, and highly efficient hybrid electrodes based on a mixture of silver nanowires and MXene (AgNW/MXene), for which **Figure** **10**a provides the fabrication process.^[^ ^146^ ^]^ The AgNW/MXene hybrid electrodes demonstrated excellent photovoltaic performance in fullerene (PTB7‐Th:PC_71_BM) and non‐fullerene (PBDB‐T:ITIC)‐based flexible OPVs. The PBDB‐T:ITIC:PC_71_BM‐based ternary OPVs with hybrid electrodes exhibited a PCE of 8.30%. Additionally, the ternary OPVs showed robust flexibility by retaining ≈85% and 91% of their initial PCEs after 1000 bending cycles at a radius of 5 and 40 mm, respectively (Figure 10b). The findings of this study pave the way for the development of new routes in the field of 2D materials for flexible optoelectronic devices.

Recently, Zhang et al. developed a semi‐transparent flexible photovoltaic supercapacitor (FPSC) system.^[^ ^147^ ^]^ Figure 10c shows the integrated flexible OPVs with Ti_3_C_2_T*_x_* as an electrode and supercapacitor with an organic ion gel (ionogel) as the device structure, while Figure 10d shows a schematic of the preparation of the flexible electrode. The ionogel was used as the electrolyte in the perpendicular direction, while the thin film of Ti_3_C_2_T*_x_* was used as a common electrode. Figure 10e shows a schematic of a flexible solid‐state supercapacitor based on the spin coating of Ti_3_C_2_T*_x_* MXene on PET sheets. The OPVs employing the Ti_3_C_2_T*_x_* electrode exhibited a PCE of over 13%. On the other hand, the supercapacitor with the ionogel electrode showed a volumetric capacitance of over 500 F cm^−3^ with enhanced stability. Consequently, the FPSC system demonstrated an outstanding storage efficiency of ≈88%, and a large average transmittance of over 33%. The FPSC system designed in this study is appropriate for printing and roll‐to‐roll manufacturing technology, which is important for realizing flexible, portable, and wearable optoelectronics. Further, **Table** **1** summarizes the key parameters of PV cells using MXenes.

**Table 1:** Summary of the key parameters of PV cells using MXenes

Absorbing layer	HTL	ETL	TCE	*V*_OC_ [V]	*J*_SC_ [mA cm^−2^]	FF [%]	PCE [%]	Ref.
MAPbI_3_:Ti_3_C_2_T*_x_*	Spiro‐OMeTAD	SnO_2_	ITO, Au	1.03	22.26	76.0	17.4	[69]
MAPbI_3_:Ti_3_C_2_T*_x_*	Spiro‐OMeTAD	c‐TiO_2_:Ti_3_C_2_T*_x_*/m‐TiO_2_:Ti_3_C_2_T*_x_*/Ti_3_C_2_T*_x_*	FTO, Au	1.09	23.82	77.6	20.1	[69]
CsFAMA‐TQD	Spiro‐OMeTAD:Cu_1.8_S	c‐TiO_2_/m‐TiO_2_	FTO, Au	1.14	24.12	78.7	21.6	[121]
MAPbI_3_	–	TiO_2_	FTO, Ti_3_C_2_	0.95	22.96	63.0	13.8	[91]
Cs_0.05_FA_0.76_MA_0.19_PbI_2.715_Br_0.285_	Spiro‐OMeTAD	MXene/SnO_2_	FTO, Au	1.11	24.34	76.4	20.6	[137]
CH_3_NH_3_PbI_3_	Spiro‐OMeTAD	Ti_3_C_2_T*_x_*	ITO, Ag	1.08	22.63	70.0	17.1	[135]
CsPbBr_3_	Ti_3_C_2_T*_x_*	TiO_2_	FTO, Au	1.44	8.54	73.0	9.01	[72]
CsPbBr_3_	–	TiO_2_	FTO, C/CNT/MXene	1.35	7.16	72.9	7.09	[143]
CH_3_NH_3_PbI_3_	Spiro‐OMeTAD	SnO_2_/MXene	ITO, Ag	1.06	23.14	75.0	18.3	[71]
(FAPbI_3_)_0.97_ (MAPbBr_3_)_0.03_	Spiro‐OMeTAD	TiO_2_/SnO_2_/ Ti_3_C_2_T*_x_*	FTO, Au	1.10	22.03	77.6	20.1	[136]
PBDB‐T:ITIC	U‐Ti_3_C_2_T*_x_*	Ca	ITO, Al	0.89	15.98	64.0	9.02	[66]
PBDB‐T:TIC	MoO_3_	UH‐Ti_3_C_2_T*_x_*	ITO, Al	0.87	17.36	60.0	9.06	[66]
PBDB‐T:ITIC	Ti_3_C_2_T*_x_*	PFN‐Br	ITO, Al	0.88	17.85	67.0	10.5	[130]
PBDB‐T:ITIC	PEDOT:PSS/Ti_3_C_2_T*_x_*	PFN‐Br	ITO, Al	0.91	17.08	70.9	11.0	[131]
PM6:Y6	PEDOT:PSS/Ti_3_C_2_T*_x_*	PFN‐Br	ITO, Al	0.83	25.63	68.4	14.5	[131]
PTB7:PC_71_BM	MoO_3_	ZnO:Ti_3_C_2_T*_x_*	ITO, Ag	0.77	17.53	69.3	9.36	[134]
PBDB‐T:ITIC	MoO_3_	ZnO:Ti_3_C_2_T*_x_*	ITO, Ag	0.93	18.63	70.3	12.2	[134]
PM6:Y6	MoO_3_	ZnO:Ti_3_C_2_T*_x_*	ITO, Ag	0.83	26.38	75.4	16.5	[134]
PM6:Y6	PEDOT:PSS	PFN‐Br	Al, Ti_3_C_2_T*_x_*	0.84	24.97	64.9	13.6	[147]
PM6:Y6	PEDOT:PSS	PFN‐Br	Ag, Ti_3_C_2_T*_x_*	0.83	24.78	64.0	13.1	[147]
PTB7‐Th:PC_71_BM	PEDOT:PSS	PrC_60_MA	MXene‐AgNw‐PUA	0.79	14.62	61.0	7.16	[146]
PBDB‐T:ITIC	PEDOT:PSS	PDINO	MXene‐AgNw‐PUA	0.86	13.98	64.0	7.70	[146]
PBDB‐T:ITIC:PC_71_BM	PEDOT:PSS	PDINO	MXene‐AgNw‐PUA	0.88	14.85	63.0	8.30	[146]
PTB7‐Th:PC_71_BM	PEDOT:PSS	LiF	Al, Ti_3_C_2_T*_x_*	0.79	14.91	65.6	7.76	[147]
P3HT:ICBA	PEDOT:PSS	PEI	ITO, Ti_3_C_2_T*_x_*	0.84	7.39	53.7	3.34	[147]
P3HT:ICBA	PEDOT:PSS	PEI	Ti_3_C_2_T*_x_*	0.84	6.91	51.5	2.96	[147]

## 5 Stability of the MXene‐Based PV Cells

In addition to the development of high‐performance PV cells, securing long‐term stability is indispensable for the commercialization of PV technology. Huang et al. measured the stabilities of PEDOT:PSS and PEDOT:PSS/Ti_3_C_2_T*_x_* composite‐based OPVs at RT in an N_2_ filled glove box.^[^ ^131^ ^]^ After 300 h, the stability of the PEDOT:PSS and composite‐based OPVs dropped to 69% and 80% of their initial PCEs, respectively (Figure 10f). The inferior stability of the PEDOT:PSS devices was attributed to the acidic nature of the PEDOT:PSS HTL, which is corrosive to ITO. The inclusion of Ti_3_C_2_T*_x_* in the PEDOT:PSS solution alleviated the corrosion tendency of PEDOT:PSS toward the photoactive layer and ITO, thus resulting in improved stability of the PEDOT:PSS/Ti_3_C_2_T*_x_* composite devices. More recently, Yu et al. measured the stability of OPVs in ambient air without encapsulation, using ZnO/Ti_3_C_2_T*_x_* composite ETLs.^[^ ^134^ ^]^ The performance of ZnO devices deteriorates severely with the decline in PCE values from 10.5% to 6.95% (PCE drop of 65%) after 40 days, owing to the infiltration of oxygen and moisture into the photoactive layer (Figure 10g). In contrast, the OPVs with the ZnO/Ti_3_C_2_T*_x_* composite ETLs retained 72.5% PCE from their original values, demonstrating relatively improved stability under the same atmospheric conditions. The improved stability of the ZnO/Ti_3_C_2_T*_x_* composite devices can be attributed to the passivation effect of Ti_3_C_2_T*_x_*, and the enhanced crystallinity of the photoactive layer.^[^ ^148^ ^]^

Furthermore, the hydrophobic nature of Ti_3_C_2_T*_x_* restricted the penetration of humidity from air into the photoactive layer. Ma et al. improved the stability of perovskite solar cells using MXene as the back electrode.^[^ ^91^ ^]^ The devices with MXene electrodes exhibited satisfactory stability with a PCE retention of over 60% after 360 h in air at RT (30% moisture), without any encapsulation (Figure 10h). The Ti_3_C_2_ layer functioned as an encapsulating layer in the devices, thus protecting the absorbing layer from moisture and oxygen. Similarly, subjecting MXene to UVO treatment also improved the stability of the devices, which was validated by Miyasaka et al. in 2019.^[^ ^135^ ^]^ The PCE of the untreated Ti_3_C_2_T*_x_*‐based devices was lowered to 30% from the original values after 384 h, whereas the devices treated with UVO for 30 min exhibited enhanced stability by retaining 70% of the PCE from the original value after 800 h. Another study on the stability of MXene‐employed PV cells was performed by Huang et al. in the same year.^[^ ^130^ ^]^ They observed a PCE retention of ≈62% from the initial value in the Ti_3_C_2_T*_x_*‐based devices, in comparison to the PEDOT:PSS‐based devices, where the PCE dropped to ≈0% after 12 h. The superior stability of MXene over the commonly used PEDOT:PSS HTL indicates its strong potential as a novel interfacial material for optoelectronic devices. **Table** **2** summarizes the function and properties of MXenes employed as different roles in PVs.

**Table 2:** Summary of the functions and properties of MXenes employed as different roles in PVs

	Functions/properties	Refs.
Additive	Enhancing the crystallinity of the perovskite materials.	[66, 70, 119, 120, 121, 122]
	Improving the conductivity and charge transfer characteristics by passivating the recombination zones.	
	Tuning the work function between the absorbing layer and interfacial layers by modifying the electronic structures.	
	Providing fine morphology control of the active layer in solution‐processed PVs.	
Interfacial layers	Excellent metallic conductivities and suitable energy level alignment at the interface by easily tunable work functions.	[66, 67, 72, 131, 132, 134, 135, 136, 137]
	High optical transmittance and large charge mobilities.	
	Increasing charge transfer paths.	
	Improving electrons and holes extraction.	
	Enhancing thermal stability.	
Transparent conductive electrodes	Suitable band alignment improves charge carrier dynamics.	[56, 62, 65, 68, 90, 91, 137, 142, 145, 146, 147]
	Enhancing the surface‐active areas, facilitating charge carrier extraction inside the PVs.	
	A promising alternative to carbon‐based expensive electrodes.	
	Providing opportunities for developing hybrid electrodes with metallic nanowires or carbon nanotubes.	

## 6 Summary and Future Challenges

The novel utilization of MXene in PV technology since the last quarter of 2018 has received immense attention from within the research community. To date, various experimental studies on PV systems that cover the electrochemical and physical aspects of MXene have been reported. This review summarized the various approaches to synthesize MXene with a comprehensive discussion of the physical, chemical, and mechanical properties of MXene. Hitherto, wet chemical treatment methods have been a well‐known and widely practiced method to fabricate MXenes. In these methods, fluoride compounds were used as etchants to obtain Ti_3_C_2_T*_x_* nanosheets from their parent phase (Ti_3_AlC_2_) by a similar approach. In other words, fluoride compounds can be used to etch layers of Al atoms under specific conditions, thereby leading to MXene layers with evolution in surface chemistry and morphology. The widely used synthesis approaches include HCl‐LiF etching and HF etching, and the chemical, physical, and electromechanical properties of Ti_3_C_2_T*_x_* are systematically discussed, because these properties may determine its future application in PV systems. Additionally, this review highlights the synergistic impact of MXene based on its various roles in PV systems.

Ti_3_C_2_T*_x_* nanosheets have been used in PV systems in different roles, for example, as an additive, as an interfacial layer material, and as a TCE. Careful design considerations and interfacial engineering are required to enhance the performance of PVs. The use of Ti_3_C_2_T*_x_* in PVs as an additive both improves the charge transfer characteristics of PVs owing to its high metallic conductivity and large mobility, and helps to optimize the energy band alignment at the interface, which leads to the enhanced performance of PVs. The WF tunability to optimize the band alignments is of great interest for enhancing the PV performance, and Ti_3_C_2_T*_x_* has the potential to be a promising candidate for tuning the WF function of PVs. In addition, the doping of Ti_3_C_2_T*_x_* in CTL heterostructures facilitates charge carrier transport through them to form a 3D conductive network and improved interfaces, owing to the enhanced conductivity and large crystal size. The unique 2D structure, exceptional hydrophilicity, excellent conductivity, and WF turnability of Ti_3_C_2_T*_x_* pave the way for the development of efficient PVs.

Although the potential for using Ti_3_C_2_T*_x_* in PV systems is remarkable, various challenges and limitations still need to be addressed to ensure their long‐term practical application in the future. The application of Ti_3_C_2_T*_x_* in PVs is still in its infancy, with studies mostly concentrating on the investigation of the viability of using various types of PVs. Additionally, previous studies incorporating Ti_3_C_2_T*_x_* in PVs have been based on experiments. Thus, simulation and theoretical studies are required to optimize the properties of Ti_3_C_2_T*_x_* terminated with different functional groups, which would validate the experimental results. Similarly, considering the toxicity of Ti_3_C_2_T*_x_* flakes and environmental safety aspects, it is essential to devise a safe and scalable synthesis route to realize Ti_3_C_2_T*_x_* in PVs for long‐term commercialization. For example, the synthesis of fluorine‐free or non‐titanium‐based MXenes without utilizing hydrofluoric acid directly or indirectly may provide safe and eco‐friendly characteristics. Likewise, achieving long‐term stability is another critical factor for PV commercialization. Nevertheless, in comparison to its counterparts in PV studies, Ti_3_C_2_T*_x_* has demonstrated relatively higher stability; however, its long‐term exposure to the ambient environment degrades the device performance, mainly because of the oxidation of Ti_3_C_2_T*_x_* flakes. The existence of defective sites on the MXene surface is a key factor in the oxidation reaction. This issue can be addressed by employing suitable surface passivation strategies that alter the oxidation kinetics. For example, polymer‐treated MXenes exhibit superior oxidation stability and mechanical strength.^[^ ^149^ ^]^ Other methods to prevent oxidation include the use of negatively charged ligands for edge capping, and sandwiching MXene films with appropriate polymer coatings.

To date, the first reported example of Ti_3_C_2_T*_x_* has been used in PVs, and ≈70 stoichiometric combinations of MAX phases and MXenes have been theoretically estimated. A few of these combinations have experimentally proven compositions, and limitless solid solutions have been discovered. For example, Mo_2_C_2_T*_x_*, Nb_2_CT*_x_*, and V_2_CT*_x_* MXenes from the M2X phase, and Cr_3_C_2_T*_x_*_,_ and Ti_3_N_2_T*_x_* from the M3 × 2 phase have demonstrated excellent physical, chemical, mechanical, conductive, and optical properties. Moreover, other than single transition metal MXenes with only one M atom, that is, Ti_3_C_2_T*_x_*, ordered double transition metal MXenes that include in‐plane ordered structures, for example, (Mo_2/3_Y_1/3_)_2_CT*_x_* (known as *i*‐MXene), and an out‐of‐plane ordered structure, namely Mo_2_TiC_2_T*_x_* (known as *o*‐MXene), have been discovered. The experimentally synthesized Mo_2_TiC_2_T*_x_* and Cr_2_TiC_2_T*_x_ o*‐MXenes were shown to have excellent semiconducting‐like and ferromagnetic properties. The most recently discovered solid solution MXene has five M layers in its structure, for example, Mo_4_VC_4_T*_x_* (M′ M″), which are expected to possess unique mechanical and electronic properties, owing to their unique structure. These MXenes, with distinctive and outstanding properties, are potential candidates for PV applications. Thus, the large and diverse family of 2D MXenes remains unexplored in PVs, which opens up the possibility of tuning the surface and structural properties of MXenes. The properties of MXenes, including the conductivity and magnetic properties, are greatly influenced by the surface terminations; for example, Nb_2_CT*_x_* terminated with Se, S, and NH groups, was superconductive, therefore modulating the single or mixed termination functional groups on the surface of MXene with selective functional groups could enhance the PV efficiencies.

MXenes with composite structures that include layer‐by‐layer, anchoring, and cross‐linked insertion may provide a diverse range of properties to be explored in PVs in the future. Likewise, the thermal and chemical stabilities of Ti_3_C_2_T*_x_* exert a strong influence on the performance of PVs, which requires further investigation. Considering that conductivity plays a critical role in improving the performance of PVs, it is necessary to create delamination procedures for MXenes (beyond Ti_3_C_2_T*_x_*) without using organic intercalants, which contribute to enhancing the conductivities and further properties of PVs. Additionally, heteroatom doping, which was previously used to enhance the performance of supercapacitors, can be utilized for Ti_3_C_2_T*_x_*‐employed PVs. Last but not least, a large‐scale controllable self‐assembly in MXene‐based PVs requires the selection of hundreds of suitable materials; thus, intelligent techniques that include machine learning, artificial intelligence, and neural networks could be exploited to realize this dream as a commercial reality. Because of the diverse properties and functionalities of MXenes, we foresee a bright future for MXene‐based PVs. This systematic review is both expected to provide a way to realize the diverse nature of MXene composites from a different perspective, and open new directions to find solutions for next‐generation PV applications.

## Conflict of Interest

The authors declare no conflict of interest.
